# Supplementary material for: Evolution and Functional Dynamics of TCP Transcription Factor Gene Family in Passion Fruit (Passiflora edulis)
Source: Plants (Basel). 2024 Sep 13;13(18):2568. doi: 10.3390/plants13182568 (PMC11435056; doi:10.3390/plants13182568)
Supplement: Supplementary file 1 [file plants-13-02568-s001.zip › plants-3152210-supplementary.pdf]

Table S1 . Number of TCP genes identified in this study and their sequence similarity with two gene-level genomic assemblies of passion fruit.

Gene Name	Gene ID	GWHAZTM00000000	%age similarity	GWHANWG00000000	%age similarity
PeTCP1	Pe1g00801				
PeTCP2	Pe2g00351	GWHPAZTM002610	98	GWHPANWG009393	100
PeTCP3	Pe2g00400			GWHPANWG006600	85
PeTCP4	Pe2g00517			GWHPANWG009194	100
PeTCP5	Pe2g01516			GWHPANWG007711	100
PeTCP6	Pe2g02083			GWHPANWG006416	80
PeTCP7	Pe2g02474			GWHPANWG006600	100
PeTCP8	Pe2g02555	GWHPAZTM000870	90	GWHPANWG016340	100
PeTCP9	Pe2g02621			GWHPANWG009194	63
PeTCP10	Pe2g02632				
PeTCP11	Pe2g03973			GWHPANWG004941	99
PeTCP12	Pe3g00812	GWHPAZTM013768	59	GWHPANWG009925	99
PeTCP13	Pe3g01513			GWHPANWG032478	100
PeTCP14	Pe3g01959			GWHPANWG026424	99
PeTCP15	Pe4g04349	GWHPAZTM004410	99	GWHPANWG003301	99
PeTCP16	Pe5g00391	GWHPAZTM009290	100	GWHPANWG000757	100
PeTCP17	Pe5g00587	GWHPAZTM009148	99	GWHPANWG029263	62
PeTCP18	Pe5g00665			GWHPANWG000523	97
PeTCP19	Pe6g00604				
PeTCP20	Pe6g01133				
PeTCP21	Pe6g02163			GWHPANWG003830	97
PeTCP22	Pe8g00389			GWHPANWG029530	98
PeTCP23	Pe8g00755	GWHPAZTM011336	99	GWHPANWG029263	98
PeTCP24	Pe8g00826			GWHPANWG029196	99
PeTCP25	Pe8g01074			GWHPANWG004327	100
PeTCP26	Pe8g02699	GWHPAZTM013768	100	GWHPANWG027966	100
PeTCP27	Pe8g03645			GWHPANWG030257	98
PeTCP28	Pe9g00054			GWHPANWG004146	99
PeTCP29	Pe9g01413				
PeTCP30	Pe9g02247	GWHPAZTM019714	85	GWHPANWG033331	100

Table S2. Ka/Ks values and estimated time of evolution among duplicated genes of TCP family in passion fruit.

Seq_1	Seq_2	Ka	Ks	My	Ka/Ks
PeTCP28	*PeTCP21*	0.08	0.52	41	0.16
PeTCP3	*PeTCP7*	0.07	0.61	48	0.11
PeTCP26	*PeTCP12*	0.23	0.60	47	0.42
PeTCP29	*PeTCP20*	0.07	0.44	35	0.16
PeTCP24	*PeTCP18*	0.19	0.63	50	0.31

Table S3. Transcriptome data of PeTCPs in different tissues of passion fruit.

Gene	Leaf	Stem	Root	Petal	Stamen	Pistil	Mature	Immature
PeTCP1	0	0.1	0	0	0	0.3	0.1	27.4
PeTCP2	6	0.1	0	0	0.3	0	0	0
PeTCP3	0.1	2.6	0.6	0	0	0.1	0	0
PeTCP4	0	0.6	0	0.4	0.1	0	0	0
PeTCP5	0.5	1.6	3.8	0.3	0.2	1.6	2.5	4.7
PeTCP6	0	0.5	1.7	12.3	14.5	1.7	0.4	13.1
PeTCP7	1.8	10.5	2.7	0.2	0.2	0.3	0	1.1
PeTCP8	1.5	0	0	0	0.5	0.2	0	0
PeTCP9	0	0.5	0	0.7	2.5	0.1	0	1.3
PeTCP10	0	0	0	0	0	0	0	0
PeTCP11	12.9	21.5	40.7	6	8.5	1.8	0.9	3.6
PeTCP12	0	0.3	0	0	0	0	0	0
PeTCP13	3.5	4.9	8.1	16.8	19.6	7.6	0.1	5.2
PeTCP14	3.1	8.6	12.6	2.2	3.1	0.7	0.8	4.5
PeTCP15	9.1	4	3.4	50.9	25.1	16.3	13.8	53.3
PeTCP16	7.7	9.3	5	10.8	5.9	5.9	0.1	2.6
PeTCP17	15.2	0.1		61.2	89.9	6.3	0	0.5
PeTCP18	0	0	0	0	0	0	0	0
PeTCP19	40.7	2.6	18.2	30.2	35.5	6.6	0	6.3
PeTCP20	5.7	5.6	18.7	5.5	8.7	0.9	0	1.4
PeTCP21	9.5	13.8	11	5.6	4.4	1.4	0.6	6.8
PeTCP22	5.7	10.2	11.5	2.1	3.6	2.6	2.6	5.2
PeTCP23	8	0.1	0	71.3	81.3	16.2	0	0.2
PeTCP24	0	0.1	0	0	0	0	0	0
PeTCP25	8.9	21	16.4	9.2	7.1	1.4	7.6	12.3
PeTCP26	0.2	0.3	0.1	0.1	0	0.2	0	0
PeTCP27	4.3	4.8	1.5	0.8	1.1	5.1	0.1	0.5
PeTCP28	2.6	7.7	13.5	1.8	0.7	0.7	0.4	2
PeTCP29	33.3	0.6	47.4	18.8	21.6	2.6	1.9	14.2
PeTCP30	24.2	2.4	17.7	8.4	7.2	0.6	3.7	5.3

Table S4. Transcriptome data of PeTCPs under hormonal treatments.

Gene Name	Controle	ABA_3h	ABA_12h	Eth_3h	Eth_12h	Auxin_3h	GA_3h	GA_12h	MeJA_3h	MeJA_12h
PeTCP1	0.1	0.3	0	0.3	0.1	0	0	0.1	0	0.1
PeTCP2	2.5	6.1	2.9	2.8	2.1	1.9	2.2	2.7	2.4	2.7
PeTCP3	2.4	2.4	0.8	1.2	0.7	3.6	0.4	0.8	0.4	1.8
PeTCP4	0	0.2	0.2	0	0.3	0.2	0.1	0.2	0.3	0.3
PeTCP5	0.7	0.3	1.2	0.9	0.8	1.5	0.7	0.2	0.8	0.2
PeTCP6	0	0	0.1	0	0	0	0	0	0.3	0
PeTCP7	23.6	8.9	11.6	11.2	12.2	34.9	11.5	14.4	12.4	9.3
PeTCP8	2.7	3.1	2	1.4	1.3	1	1.9	0.2	0.7	2.2
PeTCP9	0.1	0.1	0.2	0.1	0.1	0.1	0.1	0.2	0	0.1
PeTCP10	0	0	0	0	0	0	0	0	0	0
PeTCP11	27	12.6	18	18.6	10	31.7	14.3	13.8	7.9	23.8
PeTCP12	0	0	0	0	0	0	0	0	0	0
PeTCP13	1.4	4.8	5.4	2.9	8.5	6.2	7.7	7.2	5.9	6.9
PeTCP14	5.9	6.1	13.4	4.4	12.6	7.4	7.6	2.4	6	3.2
PeTCP15	13.6	5	23.2	1.9	17.6	2.1	19.9	10.7	24.6	6.8
PeTCP16	0.9	13.2	3.2	4.8	6.5	1.3	8.7	7.9	9.2	8
PeTCP17	17.8	38.2	46.3	38.8	56.4	36.7	27.7	52.9	26.1	34.5
PeTCP18	0	0	0	0	0	0	0	0	0	0
PeTCP19	83.6	27.9	99.2	27.5	79.7	52.4	96.3	55.9	71	43.2
PeTCP20	11.1	14.2	13.4	12.5	15.5	17.7	17.2	13.5	15.7	13.9
PeTCP21	9.8	31.7	15.9	12.9	14	27.9	13	12.2	15.2	13.8
PeTCP22	10.1	6.5	9.4	8.3	6.3	14.2	7.8	16.5	7.8	7.3
PeTCP23	8.8	26.2	5.4	10.4	3.9	8	3.3	4.4	4.5	15.9
PeTCP24	0	0	0	0	0	0	0	0	0	0
PeTCP25	18.4	11.6	15.3	5.8	21.2	21.5	21.6	12.3	14.9	15.6
PeTCP26	0	0.1	0	0	0	0	0	0.1	0	0
PeTCP27	4.7	11	4.3	5.5	9.1	21.9	10.4	6.8	5.8	13.7
PeTCP28	4.7	5.3	5.6	8.8	7.4	17.5	7.4	6.2	7.5	6.7
PeTCP29	43.5	44.8	60.7	41.8	40.6	68.7	52.8	40.2	43.2	40.3
PeTCP30	28.7	85	49.4	35.6	24.8	68.8	40.6	32.3	31.9	40.4

Table S5. Transcriptome data of PeTCPs under cold and heat treatments.

Gene	Controle	Cold_2h	Cold_3h	Cold_12h	Heat_2h	Heat_3h	Heat_12h	Heat_72h
PeTCP1	0	0.1	0	0	0	0	0.6	0
PeTCP2	6	2	1.6	4.8	0.9	0.7	1.5	1
PeTCP3	0.1	1	0.1	0.2	0.5	0.4	0.4	0.6
PeTCP4	0	0	0.3	0.4	0.6	1.9	0.1	2.5
PeTCP5	0.5	2.6	0.7	0.2	0.6	0.7	0.4	1.1
PeTCP6	0	0	0	0.1	0	0	0.5	0
PeTCP7	1.8	19.3	2.9	1.9	10.6	10.8	8.4	16.3
PeTCP8	1.5	1.6	0.4	0.9	1.5	1.6	1.6	1.8
PeTCP9	0	0	0	0.1	0.4	0.2	0.2	0.7
PeTCP10	0	0	0	0	0	0	0	0
PeTCP11	12.9	9.2	12.8	15	9	3.8	8.1	16.2
PeTCP12	0	0	0	0	0	0	0	0
PeTCP13	3.5	1	7.1	10.2	3.4	12.9	6.1	2.5
PeTCP14	3.1	5	2.4	1.6	7.1	6.4	4.3	6.3
PeTCP15	9.1	34.4	15.9	10.2	6.1	2.9	12.9	8.6
PeTCP16	7.7	1.3	28.5	35	2.3	21.7	56.9	2.5
PeTCP17	15.2	15.1	81.1	84.7	36.7	32.6	17.7	84.4
PeTCP18	0	0	0	0	0	0	0	0
PeTCP19	40.7	132.1	48.8	24.4	22.3	12	13.4	22.4
PeTCP20	5.7	10.6	14	11.2	12.5	26.5	18.1	16.1
PeTCP21	9.5	2.2	8.8	6.4	4.2	3.2	2.4	3.9
PeTCP22	5.7	14	5.6	6.2	3.6	5.2	11	5.6
PeTCP23	8	6.8	12.3	23.4	3.5	2.3	4.4	6.4
PeTCP24	0	0	0	0	0	0	0	0
PeTCP25	8.9	11.8	13	4.4	28.1	3	6.9	29.9
PeTCP26	0.2	0.1	0	0.2	0.2	0	0	0.1
PeTCP27	4.3	5.2	4.3	2.6	5.1	0.9	1.8	1.6
PeTCP28	2.6	2.6	3.9	2.3	12.3	5.8	4.6	15.8
PeTCP29	33.3	41.5	54.7	45.5	29.3	26.2	21.1	32.4
PeTCP30	24.2	13.8	36.1	39.1	29.8	33.3	32.9	42.2

Table S6. Transcriptome data of PeTCPs under salt and drought stresses.

Gene Name	Mock	NaCl_3h	NaCl_12h	NaCL_24	PEG_3h	PEG_12h	PEG_24h
PeTCP1	0	0.2	0.1	0	0	0	0
PeTCP2	6	2.3	1.7	2.8	0.9	1.7	1.1
PeTCP3	0.1	1.9	0.2	0.5	0	0.1	0.3
PeTCP4	0	0.4	0.2	0	0.1	0.6	0.2
PeTCP5	0.5	0.4	0.1	1	0.1	0	0.8
PeTCP6	0	0	0.9	0	0	0.2	0
PeTCP7	1.8	18	5.9	16.2	3.4	6.3	5.3
PeTCP8	1.5	1.9	0.5	1.1	0.3	0.1	1.7
PeTCP9	0	0.4	0	0.1	0	0	0
PeTCP10	0	0	0	0	0	0	0
PeTCP11	12.9	34.1	6.8	34.8	8.3	3.8	11.9
PeTCP12	0	0	0	0	0	0	0
PeTCP13	3.5	4	9	10	3.2	10	5.1
PeTCP14	3.1	4.9	3.4	6	1.8	0.8	7.7
PeTCP15	9.1	2.6	31.7	7.9	23.6	13.6	29.1
PeTCP16	7.7	5.3	26.9	2.4	14.6	22	10.5
PeTCP17	15.2	55.7	18.3	47.7	27.4	68.8	31.3
PeTCP18	0	0	0	0	0	0	0
PeTCP19	40.7	64.7	25.4	98.6	32.1	9	122.8
PeTCP20	5.7	13.1	13	19.8	5.9	7.2	13.5
PeTCP21	9.5	14.9	6.5	15.5	10.9	4.2	14.2
PeTCP22	5.7	13.5	10.7	16.4	6.3	6.6	7.1
PeTCP23	8	5.8	7.1	5.8	9.5	7	10.3
PeTCP24	0	0	0	0	0	0	0
PeTCP25	8.9	28.2	16.7	38	7.2	4	22.4
PeTCP26	0.2	0	0	0	0	0.1	0
PeTCP27	4.3	13.6	0	10.7	9.8	6.7	6.8
PeTCP28	2.6	8.1	2.8	8.4	1.7	1.8	3.9
PeTCP29	33.3	51.5	29	60.2	24.1	21	44.9
PeTCP30	24.2	42.2	19.5	46	22.3	8.2	37.1

Table S7. Primer names and sequences of *PeTCPs* used for qRT-PCR.

Primer name	Primer sequence
EF-1a-F	GGCCCAACTGGTCTGACTAC
EF-1a -R	TTGCGGGATCATCCTTGGAG
PeTCP1F	AGAGACCGTCATTCCAAAGTG
PeTCP1R	CCGCAAAAGCCATTCGATG
PeTcp11F	ATGGAGAGTACACAAAGCGAG
PeTcp11R	CCCATGTTGCTGTTGATGTTG
PeTcp15F	GACCCTCAAAATCCCTACCAC
PeTcp15R	AAGTCTCGCTAATGTCCACG
PeTcp16F	ATTATCTGGGTTGGAGCTTGG
PeTcp16R	TGATACATGTGGGTCAAGGC
PeTcp17F	GTCCACCTTGTCTCTGCTATC
PeTcp17R	CCTCGGATCAATCTCCAATGG
PeTCP19F	TGATTCACGAGCAGTCACAG
PeTCP19R	GCACTAGGGTCAGTATTCCATG
PeTCP23F	GATCTTCAGGATAGGCTTGGAC
PeTCP23R	GCTTGATGGTTGAGGGAAAAG
PeTcp25F	CTTAGGTTCGCTCACGTCTTC
PeTcp25R	CACCCGCTTCCCTAGTATAAAC

Table S8. Primer sequences for cloning of TCP-GFP fusion constructs.

Primer name	Sequences
35s-PeTCP19-F	tgttgacgagagctcggtaccATGGGAATGAACAGCTCGGG
35s-PeTCP19-R	tctagaggatccccgggtaccATGCTGAGAATTGGGAGAAGAAGA
35s-PeTCP1-F	tgttgacgagagctcggtaccATGGCACCAAACCCAACAAA
35s-PeTCP1-R	tctagaggatccccgggtaccGGCAAACATCTCGTTCACGG
